# Potential circadian and circannual rhythm contributions to the obesity epidemic in elementary school age children

**DOI:** 10.1186/s12966-019-0784-7

**Published:** 2019-03-07

**Authors:** Jennette P. Moreno, Stephanie J. Crowley, Candice A. Alfano, Kevin M. Hannay, Debbe Thompson, Tom Baranowski

**Affiliations:** 1 USDA/ARS Children’s Nutrition Research Center, Department of Pediatrics, Baylor College of Medicine, Houston, 1100 Bates Street, Houston, TX 77030 USA; 2 Biological Rhythm Research Laboratory, Department of Behavioral Sciences, Rush University Medical Center, Chicago, IL USA; 3 Sleep and Anxiety Center of Houston (SACH), Department of Psychology, University of Houston, Houston, TX USA; 4 Department of Mathematics, Schreiner University, Kerrville, TX USA

**Keywords:** Sleep, Circadian rhythms, Circannual rhythms, Children, School, Summer, Growth

## Abstract

Children gain weight at an accelerated rate during summer, contributing to increases in the prevalence of overweight and obesity in elementary-school children (i.e., approximately 5 to 11 years old in the US). Int J Behav Nutr Phys Act 14:100, 2017 explained these changes with the “Structured Days Hypothesis” suggesting that environmental changes in structure between the school year and the summer months result in behavioral changes that ultimately lead to accelerated weight gain. The present article explores an alternative explanation, the circadian clock, including the effects of circannual changes and social demands (i.e., social timing resulting from societal demands such as school or work schedules), and implications for seasonal patterns of weight gain. We provide a model for understanding the role circadian and circannual rhythms may play in the development of child obesity, a framework for examining the intersection of behavioral and biological causes of obesity, and encouragement for future research into bio-behavioral causes of obesity in children.

## Background

Studies examining seasonal trends in weight gain among children have found that in the US and Japan, the school year promotes improvements in weight status (i.e., decreases in body mass index (BMI), whereas children increase their BMI during the summer holiday from school [1]. Our data collected across 5 years of elementary school revealed that not all children exhibiting accelerated weight gain during the summer holiday develop overweight or obesity; however, about 18% of children begin to transition from a healthy weight to an overweight or obese status during elementary school, with most increases occurring during the summer [2]. Nine percent showed evidence of this transition during the summer holiday after kindergarten while another 9% started during the summer holiday after 2nd grade, creating a clear window for prevention efforts during early elementary school. Seasonal weight gain, driven by circannual changes in the environment (i.e., changes in the length and timing of daylight and temperature over the course of a year) is common within the animal kingdom where it confers evolutionary advantage, via preparation for winter or reproduction [3, 4]. Whether or not seasonal weight gain provided pre-industrialized humans with advantage, it currently confers evolutionary disadvantage by contributing to increasing rates of overweight and obesity [2, 5–7].

Traditional approaches to explaining seasonal fluctuations in weight gain have focused on differences in dietary and physical activity habits during in school and out of school times (i.e., school days and weekend or school holidays) [8]. The Structured Days Hypothesis [8] explains accelerated summer weight gain utilizing behavioral economic theory [9, 10] to understand how children make decisions about their time use allocations with regards to energy balance-related behaviors on structured (i.e., school days) versus unstructured days (i.e., free days when children are not in school, such as weekend or school holidays). This hypothesis proposes that the structure provided by the school year supports a healthy weight through compulsory opportunities for physical activity, regulated access to a healthy balanced diet, limited time for sedentary activities outside of school, and consistent, earlier bedtimes and wake times [8]. In the absence of similar structures during the summer holiday, children have greater autonomy over decisions related to energy balance behaviors, which may include opting for sedentary over more intensive physical activity, more calorically and less nutritionally-dense foods, and later bedtimes and wake times [8]. Implicit in this theory is that children are essentially hedonistic and when given the opportunity, they opt for less healthy options. As a result, typical obesity prevention interventions have focused on helping children to make better choices within environmental/social constraints. An almost exclusive focus on volitional control, based on a simple energy balance model of obesity, has failed to advance understanding of the potential biological and circannual causes of weight gain [11, 12]. This paper explores the role of chronobiological causes of seasonal weight gain in children and identifies potential behavioral strategies to mitigate these influences. In doing so, we take a broader perspective of human biological systems, examining the interdependence of behavior, social demands (i.e., social timing resulting from demands such as school or work schedules, social activities, community involvement, family obligations and routines, parenting practices, etc.), circadian and circannual clocks, and metabolism to consider potential mechanisms through which misalignment of these daily and annual patterns may contribute to obesity in children. Advances in the biological sciences need to inform the behavioral sciences, so that parallel complementary advances can be made.

Chronobiology, refers to the study of biological rhythms that occur in a cyclical or periodic manner, providing temporal organization to physiological processes (e.g., metabolism) with behavioral outputs of the circadian system (e.g., sleep/wake, eat/fast) [13–15]. Circadian rhythms (i.e., daily cycles of internal rhythms) occur in cycles of about 24.2 h, on average, and are entrained or synchronized primarily by exposure to the earth’s 24 h light-dark cycle. Because our circadian cycle is slightly longer than 24 h, consistent input from the light-dark cycle is needed in order to maintain a 24 h day. The body clocks are located within cells, tissues, and organs throughout the body and are organized in a hierarchical manner. At the top of the hierarchy is the central clock known as the suprachiasmatic nucleus (SCN) [16]. The SCN is primarily entrained by inputs from the light-dark cycle [17]. Similar to an orchestra conductor, the SCN uses the inputs from the light-dark cycle to determine time of day and impose temporal order to the body’s physiological functioning by sending timekeeping signals to the body’s instruments or peripheral clocks located throughout the central nervous system and the body, such as the liver, pancreas, muscle, and adipose tissue (i.e., fat) [16, 18]. Peripheral clocks in the body control physiological processes (e.g., metabolism, body temperature, hormone secretion, and immune responses) [19]. Sleep, physical activity, and eating patterns are behavioral outputs of the circadian clock. The timing of food intake entrains or synchronizes the body’s peripheral clocks [20–22], and as a result, changes in eating habits (e.g., eating late at night) can lead to misalignment of the central and peripheral clocks. Optimal functioning is dependent upon proper alignment between the light-dark cycle, the central circadian clock (i.e., SCN), peripheral clocks, and the behavioral outputs (e.g., sleep, eating, activity) [23]. Social jet lag is an example of chronic circadian misalignment that results when social demands (e.g., work or school) require individuals to live on a schedule that is not optimal for their internal rhythms, making it hard to fall asleep and wake up at socially prescribed times for school or work. This results in an unmet sleep need and an accumulated sleep debt on school or work days (i.e., social jet lag). On days with fewer obligations (e.g., weekend days), individuals compensate by waking up later, which can introduce more variability in the timing of meals, sleep, and activity patterns. The resulting misalignment between the body’s central and peripheral clocks leads to negative health outcomes, such as obesity, type 2 diabetes, cardiovascular disease, and cancer [14, 24–26].

There is growing evidence that circadian misalignment is involved in weight gain and the development of obesity [16, 19, 26–30]. Experiments in animals have demonstrated that mistimed feeding (e.g., eating during the biological night) [31, 32], a high-fat diet [33, 34], jet lag [35], and shift work [36] disrupt circadian alignment and lead to weight gain. Among humans, evidence regarding the association between circadian misalignment and obesity comes primarily from observational studies. For example, shift work is associated with an increased risk for obesity, type 2 diabetes, metabolic syndrome, and cardiovascular disease in adults, especially long-term shift workers [37–41]. Social jet lag has also been associated with obesity in adults [42] as well as adolescents and children [42–45]. Night Eating Syndrome, an eating disorder that involves eating 25% or more of one’s daily calories after the evening meal, was associated with increased risk of obesity and changes in the timing and amplitude of metabolic hormones such as glucose, insulin, ghrelin, and leptin in adults [14, 46, 47]. Finally, an association between late meal timing and weight gain or obesity has been observed in observational studies among adults [48–53] and children [54–56]. In addition, late meal timing has been shown to affect weight loss outcomes in obesity treatment interventions [57–59].

### Theoretical basis of the circadian and circannual rhythm model of accelerated summer weight gain

**Fig. 1:**
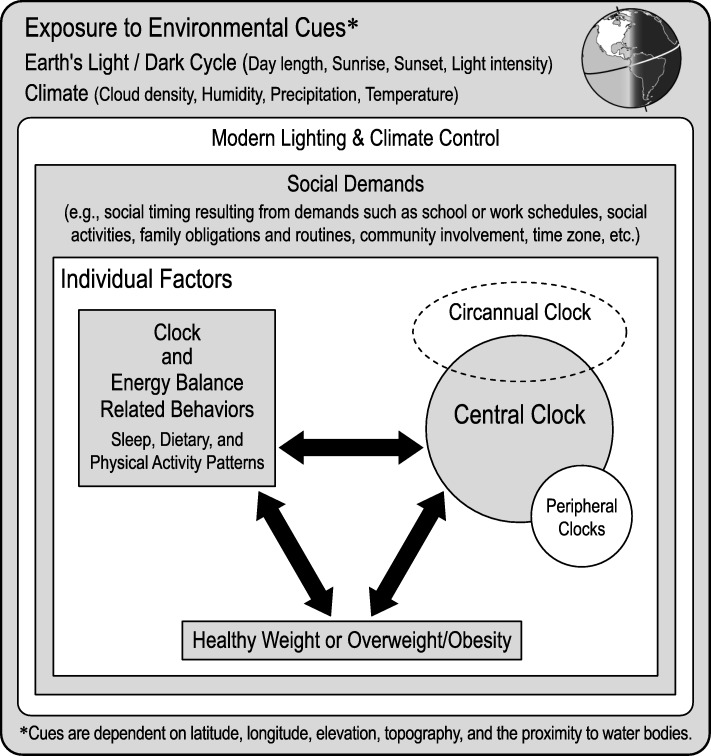
Model for Circadian and Circannual Contributions to Children’s weight gain. Figure 1 Footnote. We propose that the individual is nested within their environment which includes the influences of social demands (e.g., social timing resulting from demands such as school or work schedules, social activities, family obligations and routines, parenting practices, community involvement, time zone, etc.), the modern lighting and climate controlled environment, as well as the effect of the earth’s natural environment. Within the individual, there is an interdependence of the circadian clocks, behavior, and health. The major contribution of this model is that the circannual clock interacts with the circadian clocks to promote optimal health and disruption of children’s circannual influences may have health consequences [70]. We propose interactions within the individual and across levels of this model. For example, social demands influence an individual’s behavior which affects alignment of the clocks either by direct entrainment of the peripheral clocks (i.e., meal timing and consistency) or by affecting exposure to the light-dark cycle via sleep timing and consistency, physical activity, and exposure to artificial light at night. It is also proposed that circadian disruption caused by the school holiday may contribute to disruption of circannual rhythms of growth, resulting in accelerated summer weight gain and contributing to the development of overweight and obesity during elementary school. This model was adapted from Roenneberg T, Merrow M. The Circadian Clock and Human Health. Curr Biol. 2016;26(10):R432–443

Roenneberg developed a comprehensive model regarding the role of circadian misalignment in the development of obesity and other health conditions in which he illustrates the interdependence of the circadian clock, behavior, and health [24]. These intra-individual factors within the individual are influenced by two elements from the environment: 1) exogenous cues that synchronize circadian rhythmicity (e.g., exposure to the light-dark cycle) and 2) social demands (e.g., school or work times, social activities, community involvement, family obligations and routines, parenting practices, etc.). The major contribution of our model to Roenneberg’s model is the proposal that the circannual clock, synchronized by seasonal changes in environmental cues (e.g., light-dark cycle), also plays an important role in health outcomes (e.g., the development of obesity; Fig. 1). We propose that children exhibit a healthy seasonal pattern of weight gain and height growth that is controlled by a circannual clock. Further, we propose that the summer holiday environment is conducive to circadian misalignment based on changes in social demands leading to increases in the variability of sleep, eating, and physical activity patterns and facilitating accelerated summer weight gain. While school holiday schedules vary in timing and length around the world, many countries have adopted an agrarian school calendar offering children a summer holiday of about 6–12 weeks, typically occurring during the months of June through August in the northern hemisphere and December through February in the southern hemisphere. We hypothesize that the co-occurrence of the timing of the school holiday (i.e., conducive to circadian misalignment) during the season in which children are primed for weight gain may disrupt children’s circannual growth patterns, contributing to accelerated weight gain and the development of obesity. To support our theory, we will review the literature regarding seasonality in humans, seasonal growth in children, and biological mechanisms through which the circannual clock and circadian clock may affect the timing and velocity of children’s weight gain.

### The circannual clock

Similar to daily circadian rhythms, annual rhythms are controlled in part by exposure to earth’s light-dark cycle resulting from the 23.5° tilt of the earth on its axis, its daily rotation, and the annual orbit around the sun, as well as from climatic weather patterns [60, 61]. The effects of this tilt are most clearly seen at the extreme poles. Around the summer solstice (i.e., June 21), people living above 66.5° N in the Arctic experience 24 h of sunlight while regions below 66.5° S experience 24 h of darkness (i.e., their winter) [61]. At latitudes closer to the equator, there is less difference in day length throughout the year. The tilt not only affects day length but the ability of the sun to warm the earth. The warming effect of the sun is increased in areas of the globe receiving more direct solar radiation at any given time of the year which also coincides with a longer day length allowing for a longer warming and shorter cooling period during the night [61]. The amount of daylight a given area receives may also be determined by climatic weather patterns such as rainy and dry seasons [61].

Under natural lighting conditions (i.e., free of artificial lighting), adults exhibit a 24-h sleep-wake rhythm that is responsive to seasonal changes in the light-dark cycle [62]. The SCN (i.e., central clock) demonstrates plasticity to encode for these seasonal changes in the length of daylight, creating an internal representation of day length [63]. Information about day length is signaled to the pineal gland (i.e., area of the brain that releases melatonin) [64]. The length of melatonin release, marking the biological night, varies seasonally in response to changes in the length of the earth’s dark period [63, 65]. A bi-oscillator model of circadian regulation suggests that seasonal adaptation to the light-dark cycle is facilitated by two oscillators, one entrained (i.e., synchronized with an environmental cue such as light) via dusk, controlling the onset of melatonin and the other entrained via dawn controlling the offset of melatonin [66, 67]. The presence of two oscillators may explain individual differences in response to seasonal changes in the day length [68, 69]. Under natural lighting conditions in which the dark period is much longer in winter compared to summer, adults exhibit a longer melatonin release in winter compared to summer. However, when exposed to modern lighting conditions where dark periods vary less across seasons, adults have demonstrated a lack of seasonality in their melatonin rhythms [65], possibly representing a form of circannual disruption which may have important health consequences [70]. To our knowledge no studies have examined circadian entrainment under natural and modern lighting conditions in children. As a result, it is unclear if children lack seasonality as adults do; however, we hypothesize that the transition from the school environment to the summer holiday may be associated with changes in exposure to the light-dark cycle which may signal seasonal change to the brain.

While it is not clear whether children exhibit seasonal changes in melatonin, there is evidence of seasonality in their growth (i.e., height) [71–73] and weight gain, suggesting an endogenous rhythm of growth and weight gain in children [72–76]. Few recent studies have examined monthly changes in growth among children; however, several studies from the late 1800s to mid-1900s suggest that children tended to gain height in the spring and early summer and gain weight in the late summer and fall [73, 76]. A study of blind and sighted children living in Southern England found that sighted children demonstrated maximum gains in height between January and June, while periods of maximum growth in blind children were evenly distributed throughout the year [71]. This provides compelling evidence that seasonal variation in light-dark cycle may predict growth in children through the visual encoding of day length via the SCN (i.e., central clock). Other studies have confirmed that times of year during which light exposure is more abundant coincide with increases in height among sighted children [72, 73, 77, 78]. While the effect of the light-dark cycle on weight gain has not been tested experimentally, observational studies measuring weight on a regular basis (i.e., more frequently than bi annually) suggest maximum increases in weight tend to occur in late summer and fall when days are long, but shortening [76, 79–82]. Further studies examining seasonal patterns of growth among children in school and those not attending school have observed similar patterns regardless of school status [83]. Overall, these studies support the potential role of the circannual clock entrained by the seasonal variation in light and dark in children’s growth patterns.

### Accelerated summer weight gain

More recent studies examining the timing of increases in children’s body mass index (i.e., BMI, a ratio of height to weight) have also observed accelerated weight gain during the summer and early fall; however, this accelerated weight gain has been shown to contribute to increased rates of obesity during elementary school [1, 84]. While we concluded from our own 5 year longitudinal study that the obesogenic out of school summer environment was to blame for these findings our study design did not allow us to rule out the influence of circannual effects on children’s growth patterns [1, 6]. Increases in height in spring/early summer and increases in weight in late summer and early fall suggest maintenance of BMI, unless height gain is retarded or weight gain is accelerated. The consistent recent findings of increases in BMI during summer contributing to increased obesity rates [1, 2, 5–7, 85–88] suggest the potential contribution of disruptions to normal circannual patterns of growth. Additional studies are needed to test these hypotheses in order to clarify the potential interaction between the effects of circannual rhythms, circadian misalignment, and traditional energy balance related behaviors on children’s weight status.

### The biology underlying the association between the circannual clock and seasonal weight gain

Energy expenditure decreases during sleep and as a result it may seem counterintuitive that shortening of the sleep or the biological night would lead to weight gain [89]. However, in humans, the biological night is characterized by high levels of melatonin which plays a role in the timing of lipid oxidation (i.e., utilization of energy stored in adipose tissue), and brown fat thermogenesis (i.e., the conversion of fatty acids and glucose into heat) [89–91]. The biological day in humans is characterized by the absence of melatonin, contributing to processes involved in carbohydrate metabolism leading to lipogenesis (i.e., the creation of lipids) and storage of energy as fat in the fat cell [92, 93]. The yin and yang of the biological day and night promotes energy balance. Melatonin synchronizes metabolic function of the adipocytes for high lipolysis (i.e., fat or lipid breakdown) during the melatonin phase and high lipogenesis (i.e., lipid creation) during the absence of melatonin [92]. Melatonin also synchronizes the activation of white adipose tissue [93]. Among Siberian hamsters which do not gain weight in winter, short winter-like days led to longer nocturnal melatonin release, with greater stimulation of melatonin receptors in the forebrain, thereby engaging activation of white adipose tissue, resulting in lipolysis and a decrease in seasonal adiposity [93]. As opposed to humans, hamsters are nocturnal animals and thus melatonin release is associated with their biological day when the animal is active and feeds. Melatonin-induced browning of white adipose tissue (i.e., conversion of white adipose tissue to more metabolically active beige or brown adipose tissue) increases energy expenditure by converting fatty acids and glucose into heat, thereby increasing their thermogenic activity, resulting in weight loss [94, 95]. Greater activation of white adipose tissue due to longer melatonin rhythms resulting from longer nights during winter, may explain why children tend to not gain weight during winter and demonstrate faster weight gain during the longer days of summer [96].

#### Circadian misalignment and weight gain

Though the exact mechanism through which chronodisruption leads to weight gain is unknown, chronodisruption caused by shift work or social jet lag results in reductions in the production of melatonin [97]. Given the role of melatonin in energy balance, circadian misalignment may have important metabolic consequences due to desynchronization of processes involved in optimal energy balance [96, 98]. Among humans, even short-term misalignment of circadian rhythms with sleep/wake and fast/feed behaviors resulted in increased postprandial (i.e., after eating) glucose and insulin, decreases in leptin, and reversed the cortisol rhythm so that cortisol was high at the beginning of sleep instead of upon waking, suggesting that even short term misalignment may cause disruption of rhythms related to energy balance [36].

In addition to sleep, summer shifts in eating patterns and physical activity may also result in circadian misalignment associated with increased adiposity, mediated by the mistiming of behavioral rhythms with endogenous rhythms [16, 19, 63]. Misalignment of behavior with endogenous rhythms has been associated with changes in metabolism and development of obesity [16, 29, 30]. Physical activity has been shown to advance the circadian clock, possibly because exercise increases the amplitude of the daytime circadian and homeostatic rhythms, such as core body temperature, arousal, and sleep propensity, leading to a faster accumulation of sleep pressure (i.e., the body’s drive to sleep which accumulates as the amount of time awake increases), resulting in earlier sleep times [99, 100]. Thus, increases in physical activity could promote earlier bedtimes in children. Physical activity may also affect the central clock by maximizing outdoor light exposure as outdoor time is associated with increased physical activity [101–103], which facilitates the synchronization of internal clocks with the external environment. In addition, there is growing evidence that food synchronizes peripheral clocks such as those of the liver, pancreas, and gut [21, 22, 104]. Because humans exhibit a daily rhythm of glucose utilization with more efficient glucose utilization in the morning due to improved insulin sensitivity, followed by poorer glucose utilization and insulin insensitivity in the evening [105], eating later in the day results in acute exposure to higher postprandial blood glucose levels, with negative effects persisting through the following morning [106, 107]. In addition, shortened sleep duration is associated with a shift in melatonin rhythms, resulting in high melatonin levels in the morning upon awakening and eating the morning meal during the biological night [108], which reflects misalignment of the central and peripheral clocks. Long-term dysregulation of glucose levels may lead to alterations in caloric intake and storage which have also been attributed to shortened sleep duration [108], suggesting that the mis-timing of eating and sleep/wake patterns with endogenous rhythms may increase risk for type 2 diabetes and possibly obesity [109].

### Summary of the proposed conceptual model

Considering these findings all together, we present the conceptual model illustrated in Fig. 1. We propose that all children exhibit seasonal rhythmicity in their height and weight growth patterns which are synchronized by exposure to the earth’s seasonal light-dark cycle. A longer duration of melatonin secretion during winter nights may be associated with slower weight gain during winter, while children’s accelerated weight gain during summer may be due to a shorter duration of melatonin secretion (i.e., due to shorter summer nights). We propose that greater changes in children’s social demands during the summer holiday may lead to later and more variable bedtimes, greater exposure to artificial lighting at night, later and more variable meal times, and reduced physical activity. Variability in these intersecting daily rhythms may contribute to a blunting of circadian rhythmicity which may further reduce the amount of melatonin children are exposed to during summer (i.e. circannual rhythm disruption), thus contributing to accelerated weight gain in a manner that promotes the development of overweight or obesity during the summer holiday.

### Areas for future research

While there is evidence that children’s growth exhibits a seasonal pattern (entrained by exposure to the light-dark cycle) [71–74], the importance of the circannual clock to children’s growth and more broadly, human health, is relatively unknown. Research is needed to determine the environmental cues that synchronize circannual rhythms in adults and children (i.e., lengthening or shortening of day length, changing timing of sunrise or sunset, changes in light intensity and temperature) and how circannual rhythms vary across climates and geographic locations. Studying annual rhythms in humans is difficult due to the inability to expose humans to experimentally controlled light schedules for an entire year. However, it may be possible to manipulate environmental cues in order to determine salient synchronizers of annual rhythms at different developmental stages. For example, one study established that light therapy during winter resulted in increases in height among adolescent males during the same period, a season during which height gain typically does not occur [110]. Similar studies could be conducted to examine weight gain. We would anticipate that light box therapy would result in a shift in the circannual rhythm of growth marked by earlier timing of increased height velocity followed by earlier onset of increased weigh velocity. Further, much of what is known about human seasonality and entrainment to natural and artificial modern lighting has come from studies with adults [65, 111–113]. Children appear to be more sensitive to light exposure [4] due to more transparent ocular lenses and larger pupils [114]. As a result, studies are needed to understand how children’s circadian systems, and particularly the melatonin rhythms respond under natural lighting conditions (i.e., camping) and modern lighting conditions across seasons, geographic locations and in school and summer holiday environments. Because children are more sensitive to the effects of light [4], they may be able to maintain a circannual rhythm even when adults do not. Also the nature of the school and school holiday environment may be different enough from the typical adult office worker that it may affect their circannual rhythms in unknown ways. While it is not clear how the modern lighting environment affects children’s growth, recent studies suggest that the current environment is conducive to accelerated summer weight gain, in turn contributing to high rates of child obesity.

To examine the degree to which accelerated increases in BMI during summer are related to circadian and or circannual influences, experimental lab based studies would be ideal. However, there are many practical and ethical considerations which may limit their feasibility among children. Thus, animal models may be used to test aspects of our hypothesis such as the influence of circadian misalignment on seasonal weight gain. Observational studies measuring children’s sleep, physical activity, eating patterns, light exposure, and growth on a monthly basis and across geographic locations and cultures could explore associations between these factors. Observational studies would be instrumental in examining whether circannual rhythms in children’s growth differ by sex, ethnicity, age, and pubertal status. Such research may lead to important discoveries regarding the etiology of healthy growth and obesity in children, as well as more effective intervention tools.

Indeed, summer weight gain has not been observed across all children and critical individual factors remain poorly understood. It is possible that the effects of melatonin duration on growth are mediated solely by the circadian clocks; however, this has not yet been examined. Finally our theory of summer weight gain is not intended to replace the central role of diet and physical activity, but proposes additional elements for understanding changes in weight which are not accounted for solely by volitional increases or decreases in diet and physical activity.

### Implications for the prevention and treatment of childhood obesity

Circadian and circannual misalignment caused by changes in the timing of light exposure, sleep/wake schedules and eating patterns appear to be critical factors for unhealthy weight gain [14]. Behavioral obesity prevention interventions targeting at the out of school summer holiday environment may therefore benefit from promoting optimal circadian health during summer by encouraging consistent sleep timing on both scheduled (e.g. school) and free days, optimal duration of sleep, limiting exposure to artificial light in the evenings [115], encouraging light exposure during the day, particularly in the morning [116], encouraging physical activity (to enhance evening fatigue) [99, 100, 117, 118], promoting an overnight fast by limiting food intake in the evening [49], and maintaining consistent meal patterns [49]. It is possible that behavioral changes related to lighting exposure may be more acceptable or easily implemented than recommendations to reduce caloric intake and increase physical activity, thereby increasing rates of intervention adherence.

## Conclusion

Seasonal weight gain has been observed in children during the summer [5]. Lack of structure on free days (i.e., out of school) leading to changes in traditional energy balance related behaviors (i.e., physical activity, sedentary behavior, diet, and sleep) has been proposed to explain this common finding [8]. The Structured Days Hypothesis assumes a traditional energy balance model of weight gain, but overlooks the role of chronobiology. The mechanisms through which sleep and circadian disturbances might lead to weight loss within a traditional energy balance model are not well understood but deserving of greater empirical inquiry [119]. Later timing of sleep has been observed during periods of accelerated weight gain in children and adults, possibly due to exposure to artificial light at night, which may result in shortened release of melatonin during the biological night, thereby contributing to seasonal weight gain. Melatonin has demonstrated promise within animal and human models for the prevention of weight gain and treatment of obesity [96, 97, 120, 121]. However, melatonin rhythms may also explain seasonal weight gain in some individuals, thus having important implications for children during summer. Additional research is needed to explore this potentially important risk factor for childhood obesity. A better understanding of bio-behavioral causes of obesity will hopefully facilitate more effective prevention and treatment strategies as current strategies have been largely ineffective [122].

## Abbreviations

BMI: Body mass index
SCN: Suprachiasmatic nucleus
